# Using the multi-objective optimization replica exchange Monte Carlo enhanced sampling method for protein–small molecule docking

**DOI:** 10.1186/s12859-017-1733-6

**Published:** 2017-07-10

**Authors:** Hongrui Wang, Hongwei Liu, Leixin Cai, Caixia Wang, Qiang Lv

**Affiliations:** 1 School of Computer Science and Technology, Soochow University, 1 Shizi Street, Suzhou, 215006 People’s Republic of China; 2 Jiangsu Provincial Key Lab for Information Processing Technologies, 1 Shizi Street, Suzhou, 215006 People’s Republic of China

**Keywords:** Monte Carlo, Enhanced sampling method, Multi-objective optimization, Protein–small molecule docking, Complex structure prediction

## Abstract

**Background:**

In this study, we extended the replica exchange Monte Carlo (REMC) sampling method to protein–small molecule docking conformational prediction using RosettaLigand. In contrast to the traditional Monte Carlo (MC) and REMC sampling methods, these methods use multi-objective optimization Pareto front information to facilitate the selection of replicas for exchange.

**Results:**

The Pareto front information generated to select lower energy conformations as representative conformation structure replicas can facilitate the convergence of the available conformational space, including available near-native structures. Furthermore, our approach directly provides min-min scenario Pareto optimal solutions, as well as a hybrid of the min-min and max-min scenario Pareto optimal solutions with lower energy conformations for use as structure templates in the REMC sampling method. These methods were validated based on a thorough analysis of a benchmark data set containing 16 benchmark test cases. An in-depth comparison between MC, REMC, multi-objective optimization-REMC (MO-REMC), and hybrid MO-REMC (HMO-REMC) sampling methods was performed to illustrate the differences between the four conformational search strategies.

**Conclusions:**

Our findings demonstrate that the MO-REMC and HMO-REMC conformational sampling methods are powerful approaches for obtaining protein–small molecule docking conformational predictions based on the binding energy of complexes in RosettaLigand.

## Background

Simulating the interactions between a macromolecule and small molecule (ligand) is important for understanding the molecular basis of the mechanisms found in healthy and diseased cells [1]. The complex conformational search problem has been investigated in recent decades in order to predict the conformations of protein–small ligand docking [2]. Given the importance of conformational search, several software systems have been developed over the past 20 years, including Dock [3], FlexX [4, 5], GOLD [6, 7], Autodock [8–10], Glide [11] and others [12–14]. These software systems and sampling methods can efficiently predict realistic complex protein–ligand docking structures according to predefined sets of criteria [15]. In general, a protein–ligand docking conformational search method uses either Monte Carlo (MC) [16] search strategies or genetic algorithms [17]. However, in order to improve the sampling procedure, various advanced sampling approaches have been developed in recent years [18–20].

The MC method comprises a class of numerical methods based on random sampling and estimating the desired outputs using this sample. Integration by MC simulation evaluates *E*[*f*(*x*)] by drawing samples {*X*_*t*_,*t*=1,…,*n*} from the state space *Ω* and then approximating

1 $$ E\left[f(x)\right] \approx \frac{1}{n} \sum\limits_{t=1}^{n} f(X_{t}).  $$

Thus, the function mean of *f*(*X*) is estimated based on a sample mean. When the samples {*X*_*t*_} are independent, the law of large numbers ensures that the approximation can be as accurate as required by increasing the sample size *n*.

The replica exchange MC (REMC) method [21] implemented using independent Markov chains ${X_{n}^{i}}$(*n*≥0) is defined on the same state space *Ω* and it can be used to test several replicas in parallel in order to explore the same stationary normalized distributions *ρ*_*i*_(*x*)(*x*∈*Ω*,1≤*i*≤*N*) (due to the central limit theorem) at different “temperatures” [22, 23]. Replicas at sufficiently high temperatures are sampled broadly so the barriers will be crossed, whereas low temperature replicas can used to deeply explore the local energy minima. In the REMC method, frequent exchanges are attempted between states $X_{n}^{i}$ and $X_{n}^{j}$ of two “neighboring” Markov chains with indices *i* and *j*, which belong to different thermodynamic states, and the configurations can be identified that cross the local energy barriers more easily.

Many versions of the REMC sampling method have been used in studies related to simulation [24–26]. These search methods provide significant improvements in terms of computational efficiency compared with the traditional MC search methods. Hamiltonian [27–29] and well-tempered ensemble [30, 31] methods are used widely as MC search methods. Hamiltonian MC is a Markov chains MC method that uses the physical system dynamics rather than a probability distribution to estimate future states in the Markov chain. This allows the Markov chain to explore the target distribution much more efficiently, thereby resulting in faster convergence in *Ω*. The well-tempered ensemble can be designed to have approximately the same average energy as the canonical ensemble but much larger fluctuations. An even greater advantage is obtained when a well-tempered ensemble is combined with parallel tempering [32]. Using a well-tempered ensemble, it is possible to observe transitions between states, which would be impossible to study using the standard MC method [33]. In this study, we present novel multi-objective optimization (MO)-REMC sampling methods.

A multi-objective optimization problem (MOP) comprises several conflicting objectives that need to be optimized. In general, a MOP is defined mathematically as presented in [34].

### **Definition 1**

(General MOP): A MOP minimizes $F(\vec {x})$ = ($f_{1}(\vec {x})$, …, $f_{k}(\vec {x})$) subject to $g_{i}(\vec {x})\leq 0, i=1,\ldots,m,\vec {x}\in \Omega $. A MOP solution minimizes the component functions of a vector function $F(\vec {x})$, where $\vec {x}$ is an n-dimensional decision variable vector ($\vec {x}$ = *x*_1_,…,*x*_*n*_) from some space *Ω*, the vector $\vec {x}$ minimizes every component of $F(\vec {x})$, or at least one, and the component functions of the vector function $F(\vec {x})$ should be computable for every $\vec {x}$.

**Fig. 1:**
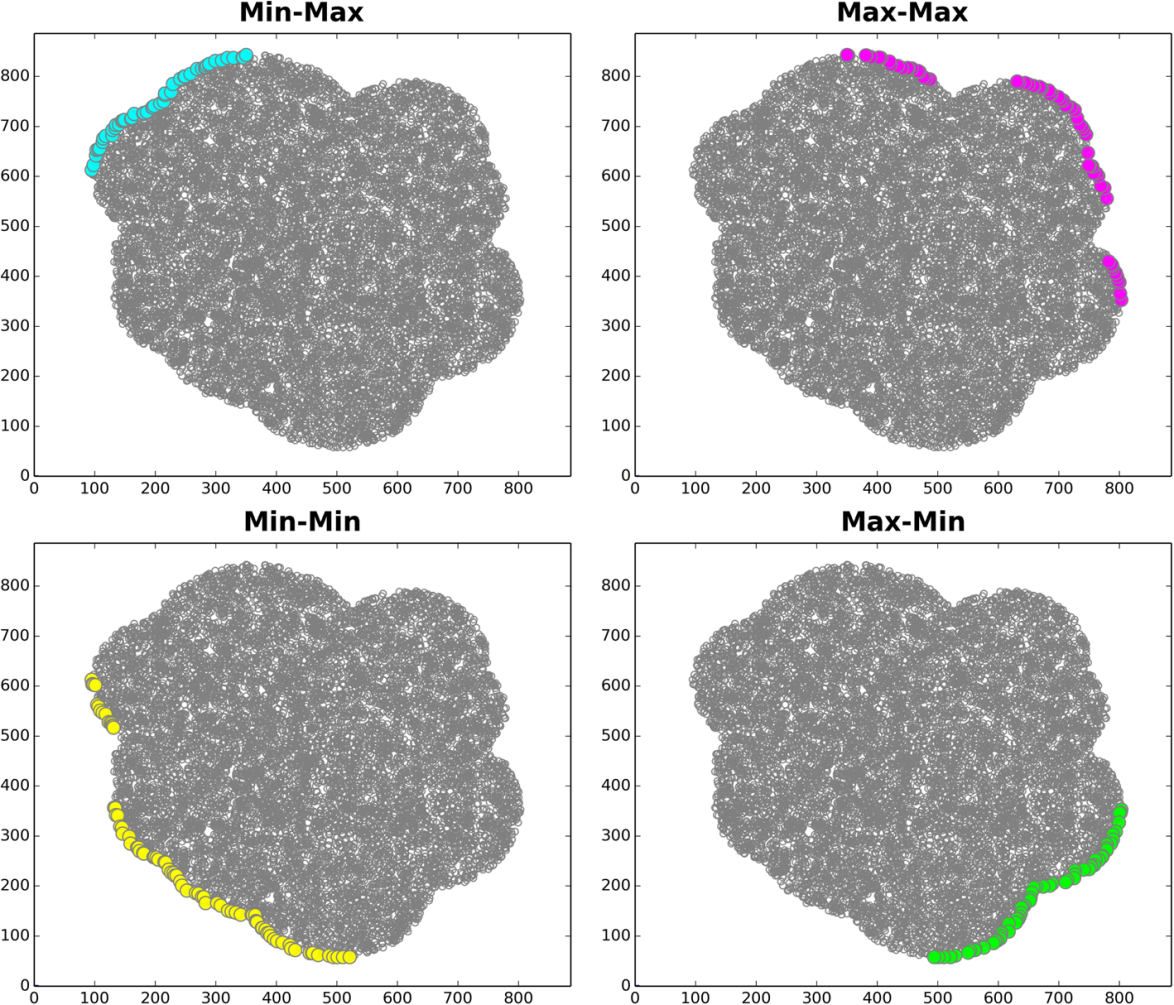
Pareto optimal solutions used to search four combinations of two objective types with discrete points

The objectives of *DEFINITION* **1** contradict each other because no point in *Ω* maximizes all of the objectives simultaneously. Thus, in order to balance them, the best tradeoffs among the objectives can be defined in terms of Pareto optimality. Using the MOP presented in *DEFINITION* **1**, the key Pareto concepts of Pareto dominance, Pareto optimality, Pareto optimal set, and the Pareto front (non-dominated solutions set) are defined mathematically as presented in [34, 35]. The multi-objective optimization approach finds the Pareto optimal set of the population, which comprises a set of solutions that are non-dominated with respect to each other. In the objective space, the set of non-dominated solutions lie on a surface known as the Pareto front. Non-dominated solution sets are those in which no other solutions are superior in terms of all attributes (objectives). Pareto optimality is effective for facilitating the convergence of the population in a low-dimensional search space [36]. By comparing every solution in the Pareto optimal set, it is always possible to improve one attribute to achieve a better gain without another becoming worse. However, each objective can be minimized or maximized when considering optimization problems with two objectives. The Pareto front approach offers a method based on attributes for finding the subset of promising solutions. This method also considers the solution attributes directly without converting them into a standard form initially. Figure 1 illustrates the case of a Pareto front with two objectives (colored points), where there is a tradeoff between minimizing and maximizing the Pareto optimal points of both the x and y coordinate values in min-max, max-max, min-min, and max-min scenarios. The scatter plots indicate the Pareto optimal set with discrete points for four different scenarios and two objectives. In each case, the Pareto optimal set always comprises solutions from a particular edge of the feasible search space for discrete points [37].

In recent studies, protein–small ligand docking prediction has focused on improving the convergence speed using sampling methods. A form of solution is used as an important component of evolutionary multi-objective optimization algorithms. It has been shown that using an elitist solution improved the convergence speed for various sampling algorithms. Therefore, in this study, we developed MO-REMC methods by using multiple non-dominated solutions as replicas for exchange during optimization at different temperatures, thereby improving the REMC sampling algorithm convergence speed associated with replica selection. We also developed methods for choosing replicas to enhance search and to improve exploration of the state space by using the Pareto front energy information. We demonstrated that the MO-REMC methods could enhance the performance of sampling methods based on a suite of benchmark test sets using the RosettaLigand protocol [38, 39]. We also performed an extensive comparative study of the proposed methods with traditional MC (detailed implementation is presented in the “Sampling methods” section in reference Algorithm 1) and REMC (see Algorithms 3 and 2) sampling algorithms based on 16 benchmark test cases. As part of this investigation, the RosettaLigand energy function total score (TScore), binding energy interface delta (IFDelta), and ligand of RMSD(Lrmsd) obtained with the proposed MO-REMC algorithms were compared with those produced by MC and REMC sampling methods, which showed that the proposed methods generally performed better than MC and REMC. The MO-REMC (see Algorithms 3, 4 and 5) and hybrid MO-REMC(HMO-REMC, see Algorithms 3, 4 and 6) methods were found to enhance the convergence to solutions compared with the MC and REMC sampling methods.

## Methods

### Test data set

The RosettaLigand protocol yielded better results with the classic MC sampling method when using a data set of 100 native protein-ligand complexes. In 71/100 cases, the lowest energy model had an Lrmsd less than 2Å [39]. We suggest that the RosettaLigand protocol cannot obtain satisfactory results in the remaining cases mainly because the MC sampling technique employed in docking is not sufficiently efficient for sampling or optimization in challenging cases. In the present study, we considered cases where satisfactory result could not be obtained with the MC approach. In all of these cases, the native complex was not recognized as a particularly low energy pose even after minimization. The 16 complexes used in this study are summarized in the “Summary of the docking results obtained using different sampling methods and scales” section.

### Preparation of the protein and ligand

A validated receptor is crucial for the successful prediction of targets. In this study, we performed repacking of the side-chain of the receptor near the initial ligand position in a similar manner to the RosettaLigand protocol [38]. Placing a ligand near clashing residues allowed the side-chains to be repacked stochastically. We generated 10 structures per receptor and the receptor structure was directly derived based on the RosettaLigand TScore to select the protein conformation with top minor TScore value. This selection process used the RosettaLigand protocol to generate 10 structures per receptor and we only selected that with the lowest energy. This procedure can resolve any pre-existing clashes between the protein side-chains and ligand, thereby gaining a large energy increase [39].

Alternatively, we treated ligand conformations as “rotamers,” which were sampled at the same time as the protein side-chains were repacked. Ligands were represented as a set of discrete conformations. To generate these conformations, all the torsional degrees of freedom in the ligand were identified and each of the torsion angles with probable conformations was compiled based on the atom type and hybridization state of the linked atoms. Next, each torsion angle was placed in one of the states considered, but conformations with internal clashes in ligand atoms were not considered, especially the conformations where the closed ring systems were not altered. Finally, we evaluated the internal ligand energy and energy minimization was applied [40]. At present, ligand conformers are generated externally in the RosettaLigand protocols. Thus, we used the Omega program (v2.3.2, OpenEye) [41] with its default settings and restrained the ligand torsions with a harmonic potential during minimization.

### Scoring function for docking

In the coarse-grained sampling stage, the coarse-grained complementary score *S*_*cg*_ is defined as

2 $$ S_{cg}=R-min(A/N,0.85),  $$

where *R* denotes ligand atoms within 2.25Å of the receptor backbone or *C* ^*β*^s (repulsive clashes), *A* denotes ligand atoms between 2.25Å and 4.75Å of any protein atom (attractive contacts), and *N* denotes the total ligand atoms. The best-scoring poses were filtered by stochastic elimination of near duplicates with a threshold of $0.65\sqrt {N}$ Å, where *N* is the number of non-hydrogen ligand atoms [39].

In the high-resolution refinement stage, the full-atom score is a linear combination of the different scoring items. These scoring items include the attractive Lennard-Jones score, repulsive Lennard-Jones score, implicit Lazaridis-Jarplus solvation score, reference energy for each amino acid, proline ring closure energy score, backbone-backbone H-bonds distant and close scores in the primary sequence, hydrogen bond energy score, probability of an amino acid at *phi* and *psi* angles, residue–residue pair probability score, and *omega* dihedral in the backbone. The high-resolution refinement scoring function *S*_*fa*_ is defined as

3 $$ \begin{aligned} S_{fa}= \sum\limits_{t=1}^{n} w_{i}s_{i},\\ \end{aligned}  $$

where *s*_*i*_ denotes different scoring items and *w*_*i*_ denotes alternative weights. The full details are described in Table 1, reference [42]. In this research, we are simply using coarse-grained sampling stage and high-resolution refinement stage scoring functions for docking, including TScore and IFDelta functions, as implemented in RosettaLigand [39].

**Table 1:** Scoring function weights used in the four sampling methods

	Weight	Weight
Score items	(Hard)	(Soft)
Proling ring closure energy	1.00	1.00
Lennard-Jones attractive	0.80	0.80
Lennard-Jones repulsive	0.40	0.60
Lazaridis-Jarplus solvation energy	0.60	0.50
Pair energy	0.80	0.50
Reference energy for each amino acid	1.00	1.00
In primary sequence		
Backbone-backbone hbonds distant	2.00	1.20
Backbone-backbone hbonds close	2.00	1.20
Hydrogen bond energy		
Sidechain-backbone	2.00	1.20
Sidechain-sidechain	2.00	1.20
Probability of amino acid at *phi* and *psi*	0.50	0.32
*Omega* dihedral in the backbone	0.50	0.50

(Hard) indicates weights used during side-chain repacking

(Soft) indicates weights used during rigid-body minimization

### Sampling methods

Our docking methods are based on the RosettaLigand(v3.4) protocol, where we use the repacking side-chain method in ROSETTA suites to generate the receptor and represent ligands as a set of discrete conformations generated by the Omega program. Finally, we examined the capability of the RosettaLigand docking protocol based on MC, REMC, MO-REMC, and HMO-REMC sampling methods.

#### MC sampling method

The MC method approximates an expectation based on the sample mean of a function of simulated random variables. The term MC generally applies to all simulations that utilize random sampling to obtain numerical solutions for a system of interest. In the general RosettaLigand protocol, MC refers to Metropolis-Hastings sampling, which samples from the Boltzmann distribution, and it was developed by Metropolis et al. in the Los Alamos team [43]. In the present study, MC simulations were performed as follows. Starting from an initial conformation of the protein–ligand interaction, a perturbation of *rotamerTrialMover*() or *packRotamersMover*() was attempted that changed the conformation of the complex. This trail *Mover*() from state last accepted (old) to state perturbed (new) is accepted based on an acceptance probability such that [39]

4 $$ prob\left[{old}\rightarrow{new}\right]:=e^{min\left(40.0, max(-40.0,\ boltz\_factor)\right)},  $$

where the *boltz*_*factor*=(*last*_*accepted*_*score*−*score*)/*k*_*B*_ *T*, *last*_*accepted*_*score* denotes the energy value of the last accepted structure of the complex, *score* denotes the energy value of the perturbed structure of the complex, *T* denotes the current temperature, and *k*_*B*_ denotes the Boltzmann constant, which is considered to be one. In order to decide whether to accept or reject the trail *Mover*(), we generate a random number, denoted by *mc*_*RG*_*uniform*, from a uniform distribution in the interval [ 0,1].

**Fig. 2:**
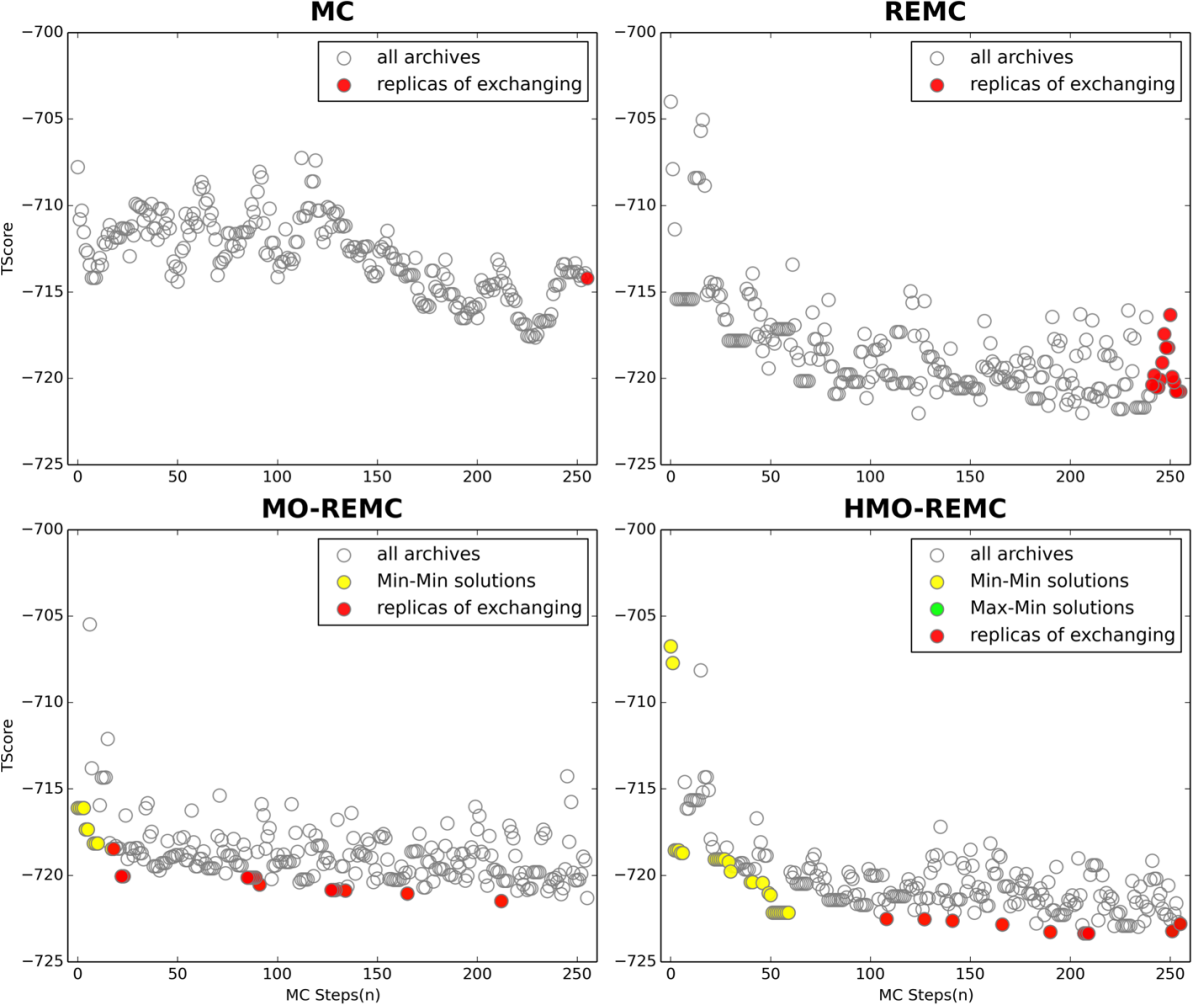
Target 2PRG replicas selected by the MC, REMC, MO-REMC, and HMO-REMC sampling methods in one iteration

Clearly, the probability that *mc*_*RG*_*uniform*[ 0,1] is less than *prob*[ *old*→*new*] is equal to *prob*[*old*→*new*]. We now accept the trail *Mover*() if *mc*_*RG*_*uniform*[ 0,1]<*prob*[ *old*→*new*] or *prob*[*old*→*new*]≥1 and reject it otherwise. The transition probability for the MC sampling method from conformation *p* to a perturbed conformation *p* ^′^ depends on the difference in *last*_*accepted*_*score*−*score* between the last accepted (old) conformation and the perturbed (new) conformation, which is determined such that

5 $$ P[{p}\rightarrow{p'}]: = \left\{\begin{array}{ll} 0, & if\ prob[{old}\rightarrow{new}] \leq mc\_RG\_uniform[0,1], \\ 1, & if\ prob[{old}\rightarrow{new}] > mc\_RG\_uniform[0,1], \\ 1, & if\ prob[{old}\rightarrow{new}] \geq 1. \end{array}\right.  $$

where *prob*[ *old*→*new*] is the acceptance probability between conformations *p* ^′^ and *p*. This rule guarantees that the probability to accept a trail *Mover*() from the last accepted conformation to perturbed conformation is indeed equal to *prob*[ *old*→*new*] [44]. If the current conformation structure is rejected, MC can retain an additional duplicate of the previous sampling structure as the sample accepted by the system. Figure 2 (left and upper panel) shows that the last sampling structure (red point) is accepted by the MC method as the exclusive solution. After many iterations, an accurate average energy value can be obtained for a complex structure. Algorithm 1 shows the pseudo-code for the RosettaLigand MC Boltzmann sampling method implementation.

In RosettaLigand, the efficiency of the MC Boltzmann sampling method can be improved by avoiding the computation of the exponential function (line 4, Algorithm 1). A more detailed interpretation is given in reference [44].



#### REMC sampling method

In current protocols, replica exchange is the most widely used method for enhancing sampling in bio-molecular simulations, where it can be viewed as a parallel version of simulation tempering, and it is also known as parallel tempering or multiple Markov chains. In the proposed method, REMC search maintains *M* identical copies of replicas as *M* sampled canonical ensembles at different temperatures. Each temperature value is unique and each of the *M* replicas has an associated temperature value (*T*_1_, *T*_2_,..., *T*_*M*_). Each of the *M* replicas independently performs a simple *MCBoltzmann*(*p,T*) search at the respective temperature setting. In addition, in our REMC algorithm, each replica *pi*′ is perturbed and the associated energy value *E*(*pi*′) is archived in ensembles *P* ^′^ and *E* ^′^. The elite replicas in the archives are selected using a procedure called *select*_*REMC*_*Replicas*(*E* ^′^,*P* ^′^). In this procedure, we select the last “*numR*” conformations that have been pushed into the queue in the archives as replicas for exchange, as shown in Fig. 2 (right and upper panels), where the last “*numR*” sampling structures are used as replicas(red points) for exchange in the REMC method. Algorithm 2 presents the pseudo-code for the selection of replicas from the archives in the implementation of the REMC sampling method.



We can represent the current state of the “*numR*” replicas selected from the archives as a protein conformation ensemble *pe* ^′^: = ($pe^{\prime }_{1}$,..., $pe^{\prime }_{numR}$), as follows, where $pe^{\prime }_{j}$ is the conformation of replica *j*, which (as stated previously) runs at temperature *T*_*j*_. During replica exchange, the temperature values of neighboring replicas are exchanged at a probability proportional to their energy value and difference in temperature. The transition probability from some current conformation $pe^{\prime }_{i}$ to a perturbed (trail *Mover*()) conformation *pei*″ is determined using the so-called Metropolis criterion, as shown in the MC sampling method section.

Exchanges are performed between neighboring temperatures, *T*_*i*_ and *T*_*j*_. The probability of an exchange depends on the energy values, *E*(*pei*′) and *E*(*pej*′), and the inverse temperatures, *β*_*i*_ and *β*_*j*_. An exchange of temperatures, and thus the relabeling of replicas, affects the state of the replica ensemble *pe* ^′^. Therefore, we define an exchange between two replicas *i* and *j* more generally as a transition from the current ensemble state *pe* ^′^ to an exchanged state *pe* ^″^. We define *l*(*pei*′) = *i*, the current label or replica number, for all $pe^{\prime }_{i}$. The probability of a transition from the current ensemble state *pe* ^′^ to an altered state *pe* ^″^ by exchanging replicas *i* and *j* is defined as [45]:

6 $$ \begin{aligned} P\left[{pe'}\rightarrow{pe^{\prime\prime}}\right]: = P\left[l(pe'_{i})\leftrightarrow{l(pe'_{j})}\right] : = \left\{ \begin{array}{ll} 1, & \Delta \leq 0,\\ e^{-\Delta}, & \text{otherwise.} \end{array} \right. \end{aligned}  $$

The value *Δ* is the product of the energy difference and inverse temperature difference:

7 $$ \Delta : = \left(\beta_{j}-\beta_{i}\right)\left(E(pe_{i})-E(pe_{j})\right),  $$

where *β*_*i*_=1/*T*_*i*_ is the inverse of the temperature of replica *i*. Potential replica exchanges are only performed between neighboring temperatures because the acceptance probability of the exchange decreases exponentially as the temperature difference between replicas increases.

The pseudo-code for Algorithm 3 illustrates the details of our REMC search procedure performed for “*numR*” replicas and a predetermined temperature range between *minT* and *maxT*. In the “ *while* *i*+1<*numR* do” loop, which runs over the pairs of replicas to be swapped, it can be seen that the swaps being attempted include pairs (0,1), (2,3), (4,5), etc., but never pairs (1,2), (3,4), (5,6), etc. This scheme will not satisfy the “detailed balance condition”(transition probabilities *i*→*j*≠*j*→*i*). Moreover, in the condition structure for *Δ*, it is obvious that the swap is rejected if *Δ* is larger than some threshold number (often 75, but also depends on the computer architecture), then the swap is rejected because *e* ^−*Δ*^ can never be larger than any random number *mc*_*RG*_*uniform*[ 0,1], and hence one call of the random number generator is saved, making the algorithm computationally more efficient.



#### MO-REMC sampling method

The REMC method involves a group of MC moves that generate a Markov chain of states. This Markov process has no dependence on history in the sense that new configurations are generated with a probability that depends only on the current configuration and not on any previous configurations. In this study, we developed the MO-REMC sampling method where the random configuration process is not Markovian so the “detailed balance criterion” is not satisfied. In contrast to the traditional REMC algorithm, which typically samples a canonical ensemble of states, we introduce a dependence on history into the REMC method and use historic multi-objective optimal Pareto front information to facilitate the selection of critical replicas of current states, which comprise a set of replicas that are similar to lower energy states but also as diverse possible. Using the generated Pareto front as representative conformation structure templates can improve the convergence of the available conformational space including possible near-native structures.

The aim of the MO-REMC sampling method is to enhance the speed of convergence for the available conformational space. The MO-REMC method employs a history-dependent Pareto frontier list to explicitly maintain a limited number of non-dominated conformations found by the REMC sampling method. Each individual in the archives generated by the REMC sampling method is evaluated using binary objectives: the sampling search steps (MC steps) and the TScore values of the perturbed conformations. The objective MC steps denote the time series for the search process and the TScore values for the perturbed conformations in RosettaLigand denote a history-dependent information map of the available conformational space. The MO-REMC sampling method is inspired by evolutionary, population-based algorithms. In the traditional REMC method, replicas at sufficiently high temperatures are sampled broadly so the barriers will be crossed, whereas low-temperature replicas can used to deeply explore the local energy minima principle. Included in multi-objective optimal method critical replicas of current states are similar greedy states, dominated non-Pareto frontier list replicas, and diverse possible characteristics. This method is effectively a combination of the REMC sampling method and historic multi-objective optimal Pareto front critical conformation structures. The experimental results show that the elite replicas generated by the historic multi-objective optimal Pareto front can enhance the speed of convergence of the available conformational space.

Algorithm 4 presents the pseudo-code for calculating the binary objectives based on the Pareto front of archives in the implementation of the MO-REMC sampling method. Each objective can be minimized or maximized according to the values of Boolean variables *maxX* and *maxY*. In this procedure, in the first step (lines 1–6), all of the solutions *x*_0_,…,*x*_*n*−1_ in the archives are the alternatives sorted in order of increasing/decreasing objective *X*, which can be minimized or maximized. Let *pf* ^′^:= {*x*_0_,*y*_0_} and *i*:=1, where {*x*_0_,*y*_0_} denotes the combination containing the first non-dominated front. In the second step (lines 8–17), for each combination in the archives {*x*_*i*_,*y*_*i*_}∈{*X,Y*}, let *pf* ^′^:= *pf* ^′^∪{*x*_*i*_,*y*_*i*_}, If {*x*_*i*_,*y*_*i*_} is not dominated by any combination according to objective *Y* that has been be minimized or maximized already in *pf* ^′^, then add {*x*_*i*_,*y*_*i*_} to *pf* ^′^. In the third step (lines 7–18), repeat from the step second until no more combinations can be added to *pf* ^′^. In the last step, iteration stops when *i*=*N*, where *N* denotes the number of combinations in the archives.



In addition, in the middle of each iteration of the MO-REMC sampling method, a set of conformations is provided instead of the last set of conformations using the *select*_*MO*−*REMC*_*Replicas*(*E* ^′^,*P* ^′^) procedure, whereas the REMC sampling method uses *select*_*REMC*_*Replicas*(*E* ^′^,*P* ^′^). The *select*_*MO*−*REMC*_*Replicas* function is obviously designed to select the conformations from the archived and the last “*numR*” min-min scenario Pareto optimal solutions set that are non-dominated relative to the other conformations, as shown in Fig. 2 (left and lower panel), where in the last circle, the last “*numR*” sampling structures are used as replicas(red points) for exchange in the MO-REMC method, and the min-min scenario Pareto optimal solutions set is denoted by yellow points (partial points are covered by red points in Fig. 2). These min-min scenario Pareto optimal solutions from the archives provide a natural and rapid convergence source, which is used to obtain alternative comparison sets from the archives. The pseudo-code in Algorithm 5 describes the procedure for determining whether to accept or reject the Pareto front, as well as for deciding whether to select replicas for exchange or not.



#### HMO-REMC sampling method

The pseudo-code of our implemented method for selecting HMO-REMC replicas is presented in Algorithm 6. We experimented using this variant of the MO-REMC algorithm with 16 protein–small ligand docking cases, which differed only in terms of the procedure used for selecting elite solutions in the MO-REMC sampling method. Updating of the replicas occurs in the MO-REMC method, which ensures that it only contains non-dominated solutions where both the objective MC steps and TScore can be minimized. Thus, the replicas for exchange cover a diverse range of individuals so the min-min scenario non-dominated solutions assigned to replicas truly reflect the quality of the MO-REMC sampling method. The MO-REMC sampling method exclusively uses replicas from the archives where both the objective MC steps and TScore are minimized.



Similarly, in the HMO-REMC sampling method, the replica selection method is based on the solutions in the archives where the non-dominated solutions from both the objective MC steps and TScore are minimized, as well as the maximized objective MC steps and minimized objective TScore values. Figure 2 (right and lower panel) shows that lower energy non-dominated solutions are used in min-min and max-min scenarios Pareto optimal solutions as replicas(red points) for exchanging in the HMO-REMC method. The min-min scenario Pareto optimal solutions set is denoted by yellow points and the max-min scenario Pareto optimal solutions set by green points. Obviously, the replicas do not include all of the lower energy non-dominated solutions in the MO-REMC sampling method. Our MO-REMC variant, the HMO-REMC sampling method, uses hybrid non-dominated solutions to select the solutions where both the objective MC steps and TScore are minimized, as well as the maximized objective MC steps and minimized objective TScore non-dominated solutions. In particular, in each replica selection step, all the lower energy non-dominated solutions in both the min-min and max-min scenarios will be used preferentially as replicas for exchange. If the number of solutions is less than *numR*, which is the number of replicas used for exchanging, the non-dominated solutions set is hybridized, where both the min-min and max-min scenarios non-dominated solutions are used iteratively to fill the replica set in order of the TScore value sequence. Replica selection in the MC, REMC, MO-REMC, and HMO-REMC sampling methods is illustrated in Fig. 2.

### Implementation in Rosetta

All versions of our MC protein–ligand docking sampling methods were coded in C++ and compiled using g++ (GCC v4.4.7). Algorithm 1 presents the pseudo-code to illustrate the details of our MC search procedure for a single replica with *N* times MC runs (*N*=*numR*×*numC*) and a predetermined number of temperatures (*T*=2.0). Algorithm 3, presents the pseudo-code for the implementations of our REMC sampling methods. In order to demonstrate the effectiveness of the REMC algorithms, including REMC, MO-REMC, and HMO-REMC, and without prior knowledge of the problem instances, we fixed the parameter configuration in all of the experimental cases (*numR,numC,repackNth,minT,maxT*) : = (16,16,5,2,4), where *numR* is the number of replicas simulated, *numC* is the number of local circle steps in REMC search, *repackNth* is the number of iterative steps performed by a *packRotamersMover*() mover, and *minT* and *maxT* are the minimum and maximum temperature values, respectively. All versions of our REMC algorithms were run on 16 processors and they were parallelized.

Multiple independent trajectories were used to generate an ensemble of docking models near the native complex using the MC, REMC, MO-REMC, and HMO-REMC sampling methods. In all of the tests in this study, we performed 5000 docking trajectories (runs), 16×16×5000 MC steps, for each receptor–ligand pair in the predictive structures, which required 30–50 processor-hours on a 1.9 GHz CPU and 2 GB memory per core Linux cluster. The results of these docking calculations were typically evaluated based on the “energy *versus* rmsd” plot where IFDelta scores were plotted *versus* Lrmsd values, and the effectiveness of each sampling method was judged according to the “funnel-like” character of the plot. In this procedure, we first discarded any structures where the ligand was not touching the protein (scoring function item ligand_is_touching=0). Second, we took the top 5% of structures based on the total energy. Finally, we ranked the remaining decoys based on the RosettaLigand IFDelta between the protein and ligand. We obtained better results with these ranking scheme and parameters.

## Results and discussion

### Comparison of different sampling methods

**Fig. 3:**
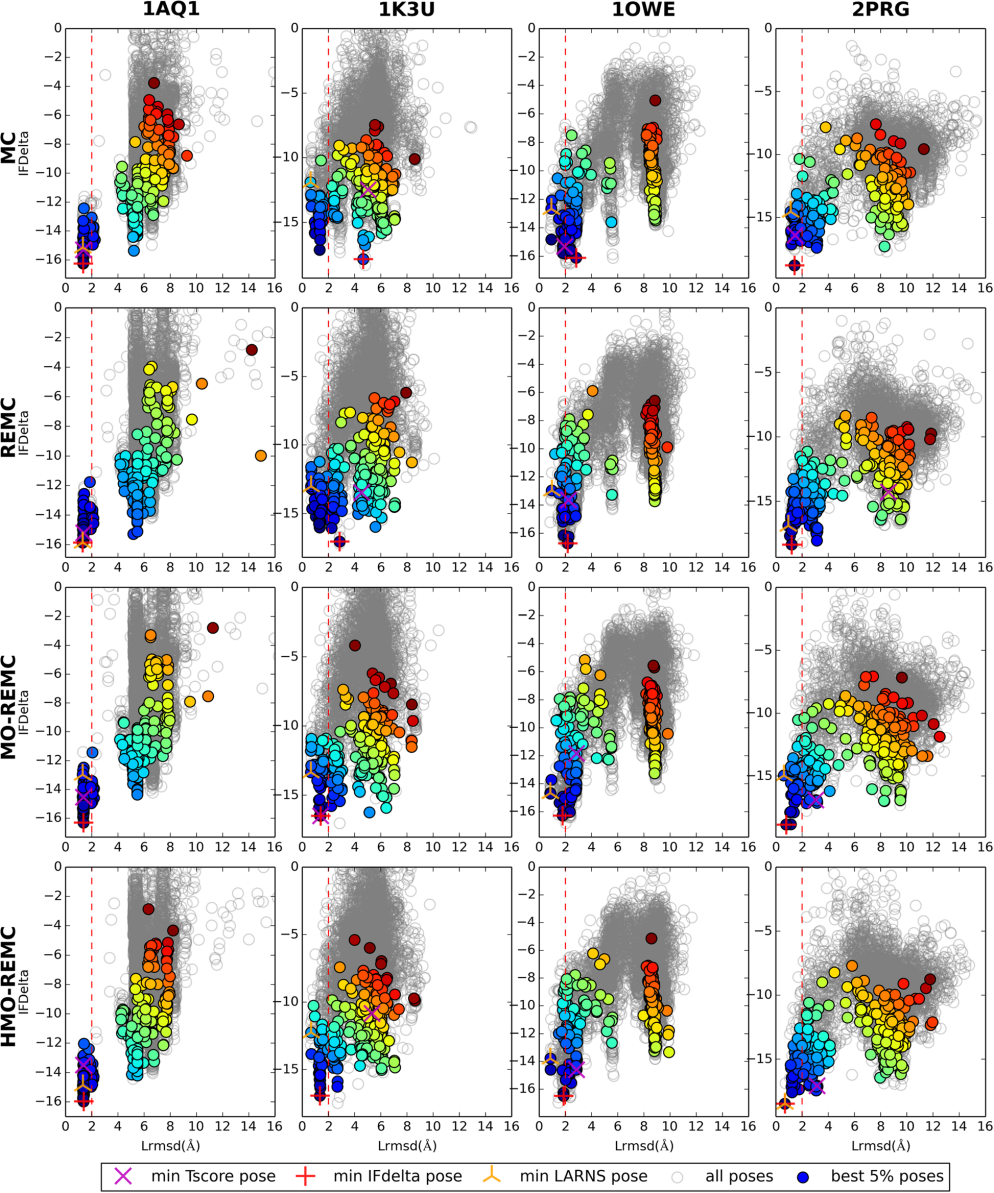
Docking results for the 1AQ1, 1K3U, 1OWE, and 2PRG targets using the four sampling methods after 1.28 ×10^6^ MC steps

In the procedure using different sampling algorithms, for each crystal structure target in the test data set, the ligand was extracted from the native complex and re-docked into the binding pocket. The Lrmsd value was calculated between the predicted positions *C* ^*α*^ of the ligand and the ligand *C* ^*α*^ in the experimental crystal structure, and Lrmsd ≤2Å was used as the criterion for success. Using the classic MC sampling method, the protein included backbone translation and rotation as well as repacking of the side-chain of the receptor, and we only selected the lowest pose in terms of energy with the traditional RosettaLigand docking protocol. As shown in Fig. 3, for the 1K3U, and 1OWE targets, the MC sampling method could not produce better experimental binding poses for the ligand in these complexes compared with those reported previously [39] even after 1.28 ×10^6^ MC steps. For 1K3U, and 1OWE, the docking results did not satisfy the requirement in terms of Lrmsd ≤2Å, but they converged based on “IFDelta *versus* Lrmsd,” as shown by the “funnel-like” character of the plot at the lower left. Successful predictions were made for the 1AQ1 and 2PRG targets using the MC sampling method, but the predictions were not sufficiently good for all of the target protein structures using the four sampling methods (see the docking results obtained using the REMC, MO-REMC, and HMO-REMC sampling methods in the figure).

The aim of REMC sampling methods is to increase the scope and depth of sampling by exchanging configurations between replicas characterized by slightly different temperature parameters. The REMC sampling method has been employed widely to enhance sampling methods by crossing energy barriers and accelerating the convergence of MC simulations. For a specific target, the MC sampling method may not be sufficient to cover some important regions of the conformational space that can be recognized by a number of ligands. However, enhanced sampling methods such as REMC, MO-REMC, and HMO-REMC can be used to generate a large number of receptor conformations for protein–ligand docking. Thus, in this study, in order to sample more of the receptor backbone and side-chain flexibility in each case, we tested 5000 decoys with each enhanced sampling method and only selected the lowest energy pose from these trajectories based on the IFDelta function as implemented in RosettaLigand [38, 39]. As shown in Fig. 3, the RosettaLigand protocol based on the REMC method obtained the lower energy pose (1OWE), faster convergence of the lower energy pose (2PRG), cross-local energy minima (1K3U), and the binding poses of the alternative ligand for the first pose within 2Å Lrmsd. By contrast, for 2PRG, the MO-REMC and HMO-REMC sampling algorithms obtained nearly perfect results within 1Å Lrmsd as well as faster convergence for more of the predicted structures with the lowest IFDelta scores.

### Comparison of different sampling scales

**Fig. 4:**
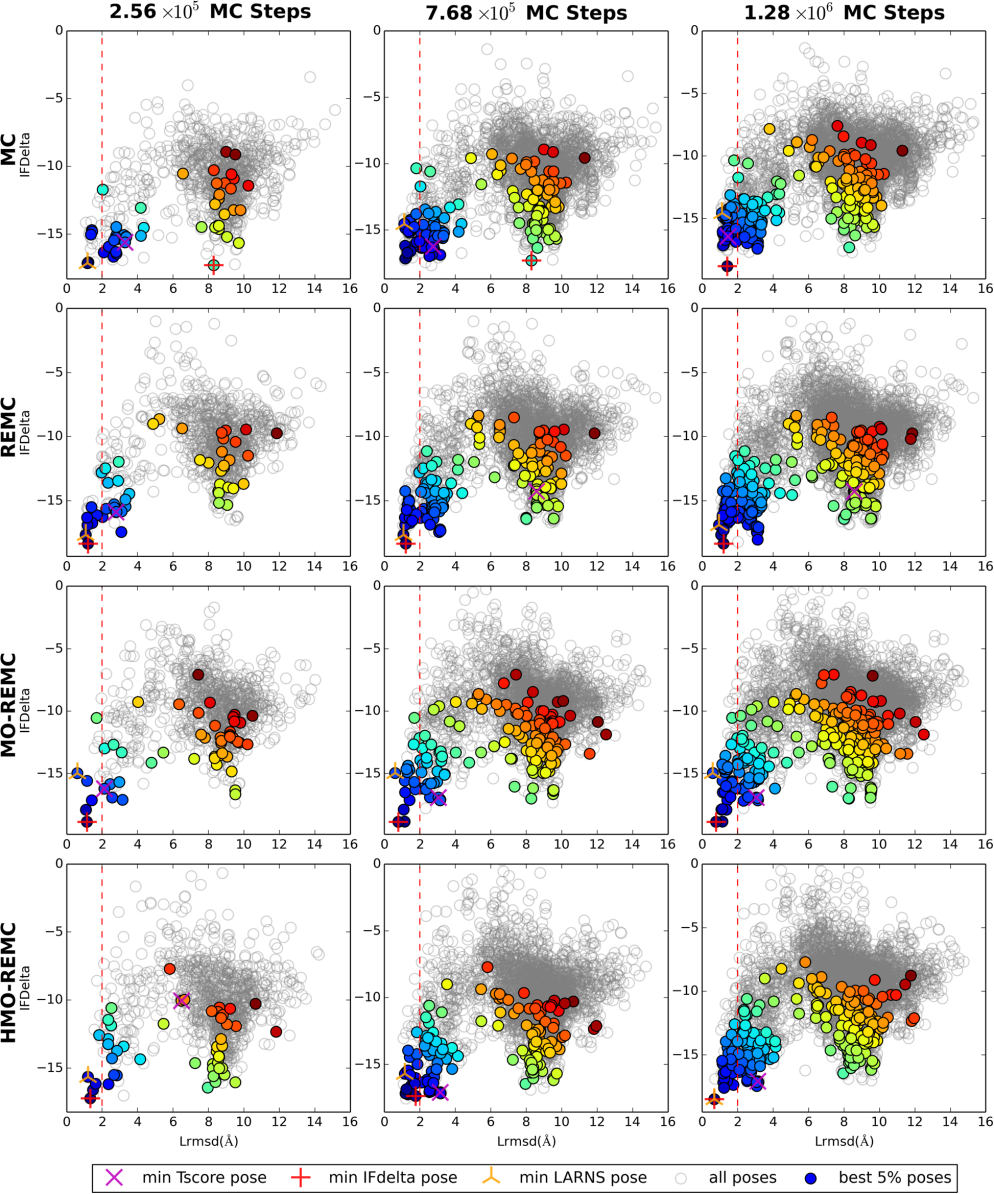
Docking results for the 2PRG target using the four sampling methods after 2.56 ×10^5^, 7.68 ×10^5^, or 1.28 ×10^6^ MC steps

The evolution of sampling in terms of the IFDelta and Lrmsd scores with different sampling scales is shown for one representative target (2PRG) in Fig. 4. For 2PRG, the four sampling methods could progressively sample lower (more favorable) IFDelta values as the number of MC steps increased from 2.56 ×10^5^ to 1.28 ×10^6^. However, the enhanced sampling methods obtained faster convergence in terms of IFDelta, as well as the HMO-REMC method compared with the MO-REMC method for Lrmsd <=2Å. The MC sampling method successfully sampled solutions with Lrmsd <=2Å after 1.28 ×10^6^ steps, whereas the REMC, MO-REMC, and HMO-REMC sampling methods could reach near-native solutions, particularly the MO-REMC method, which obtained Lrmsd <1Å solutions after only 7.68 ×10^5^ MC steps. In terms of the IFDelta scores, after 1.28 ×10^6^ MC steps, the MC sampling algorithm successfully sampled near-native solutions with Lrmsd of 1.42Å and the IFDelta score value was –18.8. By contrast, after only 2.56 ×10^5^ MC steps, the REMC, MO-REMC, and HMO-REMC methods obtained Lrmsd scores within 1.20Å, 1.14Å, and 1.33Å, respectively, and the IFDelta scores were –18.4, –18.9, and –17.2, respectively. Furthermore, after 1.28 ×10^6^ MC steps, the three enhanced sampling algorithms sampled near-native solutions with Lrmsd scores of 1.20Å, 0.79Å, and 0.69Å, respectively. In addition, the IFDelta scores converged around –18.6 ±0.3. Similar trends were also observed in all the other test cases.

### Summary of the docking results obtained using different sampling methods and scales

**Fig. 5:**
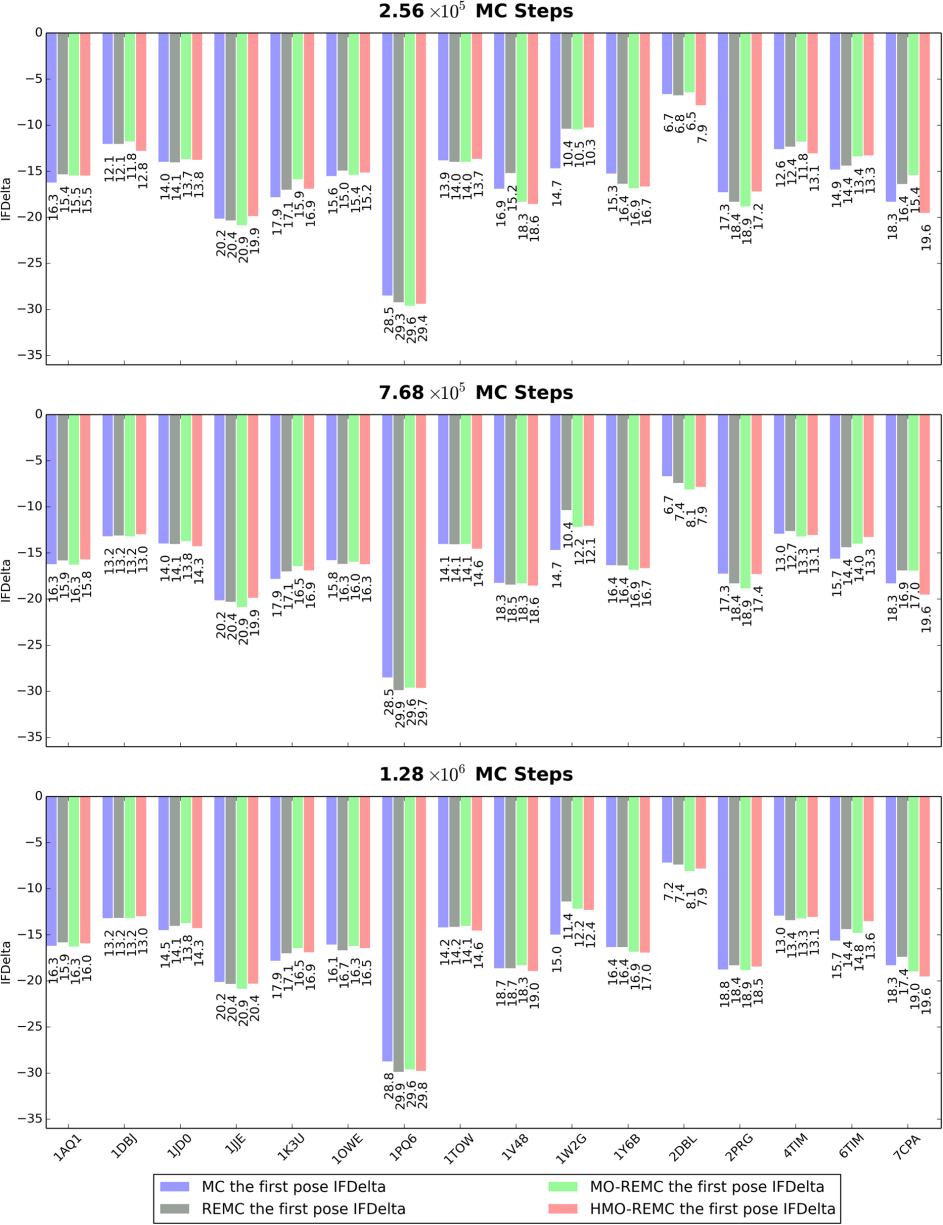
Summaries of the IFDelta scores with the MC, REMC, MO-REMC, and HMO-REMC sampling methods at three sampling scales

In general, better docking results are achieved by sampling lower docking score value conformations. So, the first parameter that we evaluated was the global performance of the docking results in terms of the IFDelta score. For all 16 cases, the evolution in terms of IFDelta using different sampling scales in the four sampling methods is shown in Fig. 5. As shown by the histogram of IFDelta values for the 16 individual targets, the four sampling methods could sample near-native docking solutions with more negative IFDelta scores at three sampling scales in 2.56 ×10^5^, 7.68 ×10^5^, and 1.28 ×10^6^ MC steps. However, using the same number of MC steps (2.56 ×10^5^, 7.68 ×10^5^, or 1.28 ×10^6^), the enhanced sampling methods could sample solutions with lower IFDelta scores than the classic MC sampling method. The MO-REMC, and HMO-REMC enhanced sampling methods obtained better docking results in 9/16 cases (1AQ1, 1DBJ, 1JJE, 1TOW, 1V48, 1Y6B, 2DBL, 2PRG, and 7CPA) with lower final IFDelta scores compared with the standard MC and REMC sampling methods after 1.28 ×10^6^ MC steps. It was interesting that the MO-REMC sampling method obtained better docking results in 7/16 cases, with lower IFDelta scores compared with the HMO-REMC sampling method. However, in 3/16 cases (1OWE, 1PQ6, and 4TIM), the REMC method obtained configurations, which were closer to the lower binding energy form compared with the MO-REMC methods. By contrast, the MC sampling algorithm succeeded also in the cases of 1JD0, 1K3U, 1W2G, and 6TIM after 1.28 ×10^6^ MC steps. The results based on the 16 test cases indicate that the MO-REMC and HMO-REMC enhanced sampling methods performed better than the MC and REMC sampling methods. The results also showed that the IFDelta values could vary dramatically for different targets and sampling methods, whereas the IFDelta scores obtained for the same target with the REMC, MO-REMC, and HMO-REMC enhanced sampling methods varied only slightly. For example, for 1PQ6, the MC method achieved an IFDelta value of around –28.8, whereas the REMC, MO-REMC, and HMO-REMC sampling methods obtained a binding energy value of around −29.8±0.2. In addition, for 1DBJ, the four sampling methods achieved similar IFDelta scores of around 13.2±0.2 after 1.28 ×10^6^ MC steps.

**Fig. 6:**
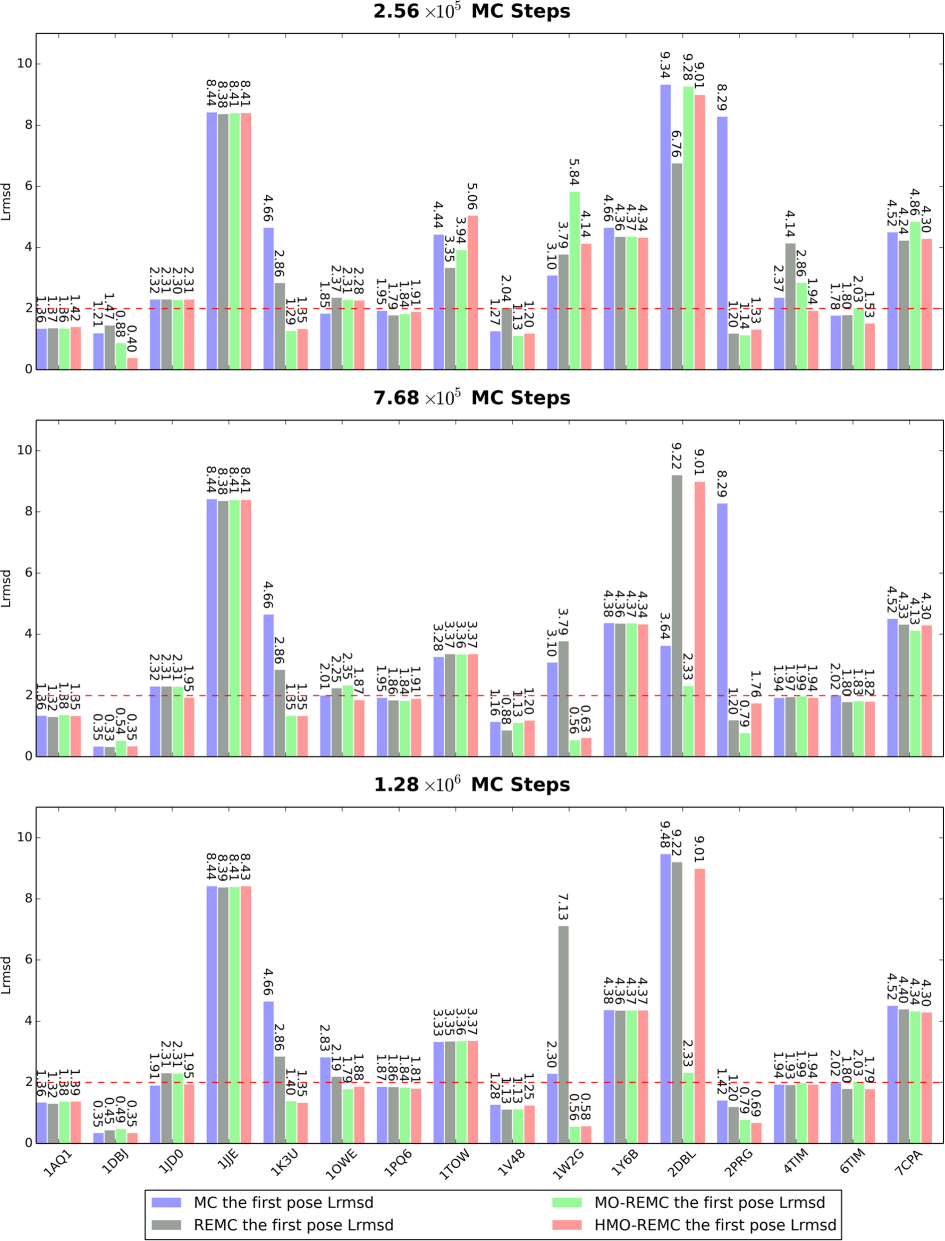
Summaries of the Lrmsd values with the four sampling methods after 2.56 ×10^5^, 7.68 ×10^5^, or 1.28 ×10^6^ MC steps

The second parameter that we analyzed was the overall performance of the docking results in terms of the Lrmsd value. An overview of the Lrmsd values obtained for the individual targets is shown in Fig. 6 at three sampling scales of 2.56 ×10^5^, 7.68 ×10^5^, or 1.28 ×10^6^ MC steps. For each target, the Lrmsd values are presented after docking the ligand into alternative receptor structures using the MC, REMC, MO-REMC, and HMO-REMC sampling methods. For each of the 16 targets, the bars from left to right correspond to the results for the protein based on the MC, REMC, MO-REMC, and HMO-REMC sampling methods, respectively. For 1OWE, and 1W2G, the MC sampling method achieved better (slightly) solutions within 2Å Lrmsd compared with the enhanced sampling methods after 2.56 ×10^5^ MC steps. However, for 14/16 cases, excluding 1JD0, and 1TOW, using the enhanced sampling methods achieved better minimum Lrmsd values for docking with the protein than the MC sampling method after 1.28 ×10^6^ MC steps. In particular, for 1W2G, and 2PRG, the MO-REMC enhanced sampling method obtained Lrmsd values that were close to the perfect results within 1Å Lrmsd. These results have never been obtained before using MC sampling methods, and we showed that the MC sampling method could not obtain satisfactory samples of complicated protein flexibility after 1.28 ×10^6^ MC steps. Finally, the Lrmsd values for individual protein structures could vary dramatically using different sampling methods and they changed greatly after 1.28 ×10^6^ MC steps, thereby suggesting that in structure-based protein–ligand docking experiments, different sampling methods can significantly affect the docking results in terms of both depth and breadth. For example, for 2PRG, the best performing target obtained using the MC sampling method only achieved an Lrmsd value of around 8.29Å after 7.68 ×10^5^ MC steps, whereas the REMC enhanced sampling method gave an Lrmsd value within 1.20Å, but the MO-REMC and HMO-REMC sampling methods obtained the best docking results within 0.79Å and 1.76Å Lrmsd, respectively.

### Convergence with different sampling methods

**Fig. 7:**
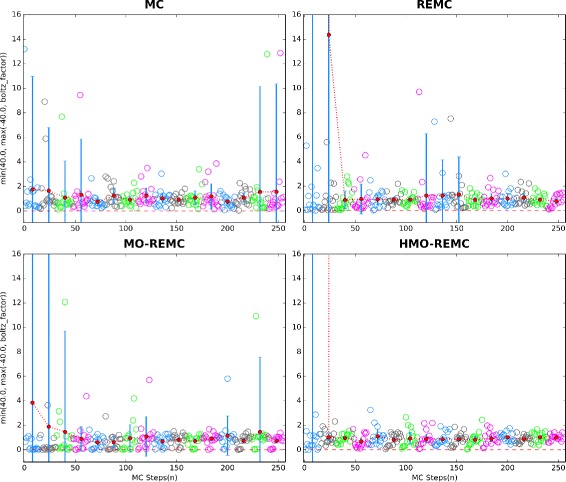
For 2PRG, the probability of finding each replica at different temperatures

Next, we briefly discuss how different sampling methods can affect the rate of convergence. Firstly, in order to demonstrate that the MO-REMC and HMO-REMC sampling methods proposed here provide an efficient sampling technique in temperature space, we calculate the probability of finding each replica at different temperatures. For the RosettaLigand docking protocol, the probability value with energy *score* in heat capacity and temperature *T* is described by Eq. (4), but no exponential calculation. In Fig. 7, for target 2PRG, we show that using the MC, REMC, MO-REMC, and HMO-REMC sampling methods, the probability of finding each replica at different temperatures progressively flattened over *numR*=16 replicas simulated through *numC*=16 local circles(*numR*×*numC* MC steps). On each sub-figure, the red circle points correspond to the probability average values of *numR*=16 replicas simulated through *numC*=16 local circles. The results obtained by the MC sampling method show that after *numR*×*numC* MC steps, the probability average values converged slowly to 1.56 with a wider fluctuation variance value of 8.78. However, using the enhanced sampling methods, REMC, MO-REMC, and HMO-REMC, the results show that the probability average values converged faster to 0.78, 0.73, and 0.99, with a narrow margin fluctuation variance value of 0.22, 0.18, and 0.07, respectively. Especially, for the HMO-REMC sampling method, the probability values of finding each replica at different temperatures show a fairly flat probability distribution. The probability results show that a strong temperature dependence of energy for complex protein–ligand docking systems.

**Fig. 8:**
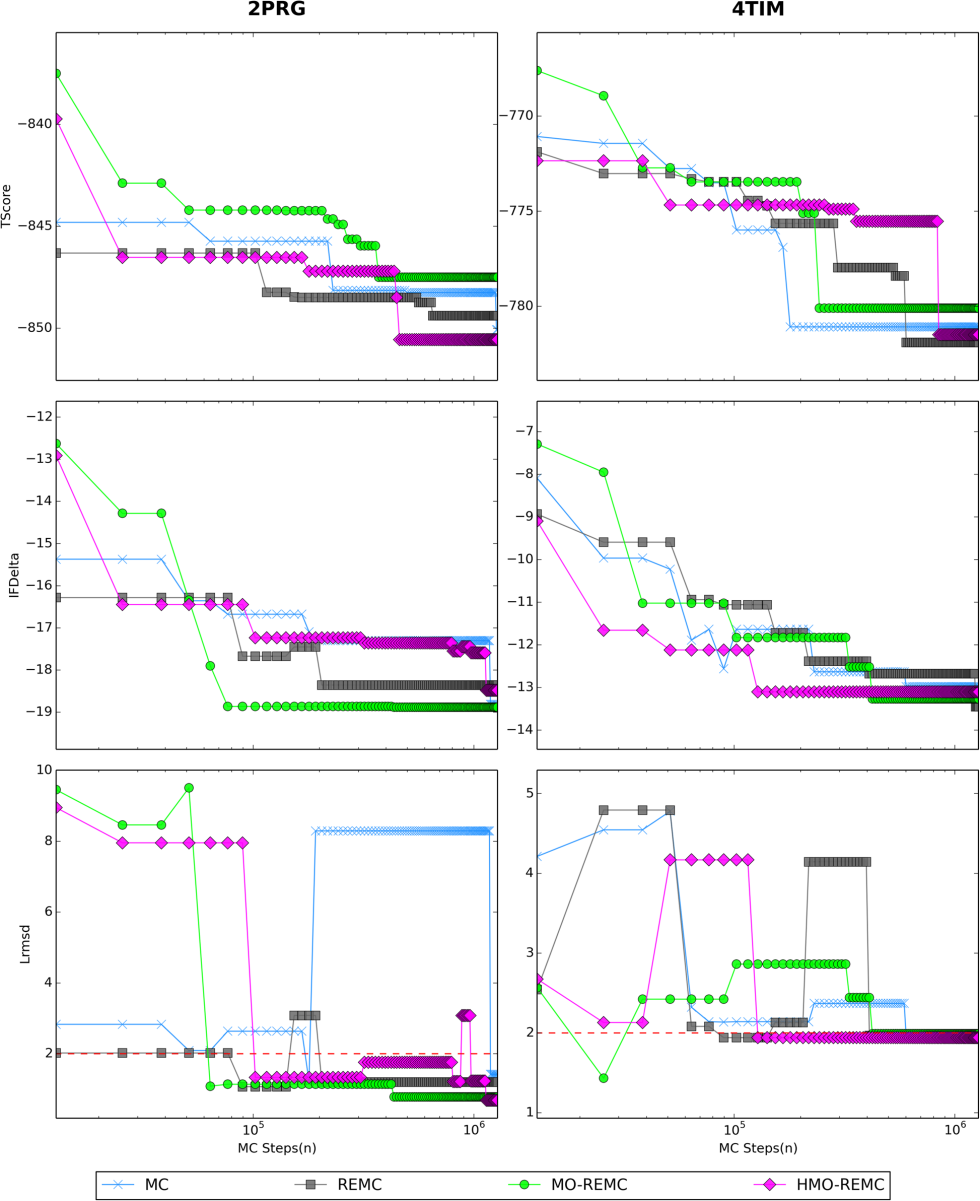
Convergence of TScore, IFDelta, and Lrmsd *vs*. MC steps for the 2PRG and 4TIM targets

Secondly, in Fig. 8, for the 2PRG and 4TIM targets, we show how the estimated TScore and IFDelta scores obtained using the MC, REMC, MO-REMC, and HMO-REMC sampling methods converged over a simulation of 1.28 ×10^6^ MC steps. For comparison, we also show the same Lrmsd values calculated after 1.28 ×10^6^ MC steps. For 2PRG, the results obtained by the enhanced sampling methods are shown that after 7.68 ×10^5^ MC steps, which demonstrate that the IFDelta score converged almost exactly to –18.2, with small fluctuations in the order of ≈0.8. However, using the classic MC sampling method, almost all of the 7.68 ×10^5^ MC steps were required to obtain a converged result with an IFDelta value in the order of –17.3, as shown in Fig. 8 (left and middle panels). After 1.28 ×10^6^ MC steps, however, four sampling methods could obtain better convergence in terms of IFDelta, TScore, and Lrmsd. This represents an improvement in the sampling efficiency by one order of magnitude and it is very likely that this could be improved further, such as by incorporating information from the Hamiltonian. One of the most important tests of convergence for a protein–ligand interaction when sampling a complex transformation is the sensitivity of the results to different sampling methods. Thus, to exclude any dependence on different sampling methods, we also calculated the Lrmsd values for the four sampling methods after 1.28 ×10^6^ MC steps and found that the estimated Lrmsd values agreed very well with the results based on the IFDelta values. These results are presented in Fig. 8 (left and lower panel). To facilitate a comparison with other targets, we also performed sampling for 4TIM using the four sampling methods, as shown in Fig. 8 (right panels), which clearly demonstrate that running 1.28 ×10^6^ MC steps for 4TIM was sufficient to obtain a converged estimate of the IFDelta score. In particular, using the MO-REMC sampling method, the estimate of the IFDelta score converged rapidly. However, the MC sampling method might obtain better convergence in terms of IFDelta and TScore as well as Lrmsd, but the rate of convergence was slower. The REMC sampling method achieved better convergence after 1.28 ×10^6^ MC steps, but the results indicated that the convergence rate was slower than that using the MO-REMC and HMO-REMC sampling methods in terms of speed and depth. In addition, the HMO-REMC sampling method performed slightly better than the MO-REMC sampling method in 9/16 cases after 1.28 ×10^6^ MC steps, as shown in Fig. 6 (lower panel).

**Fig. 9:**
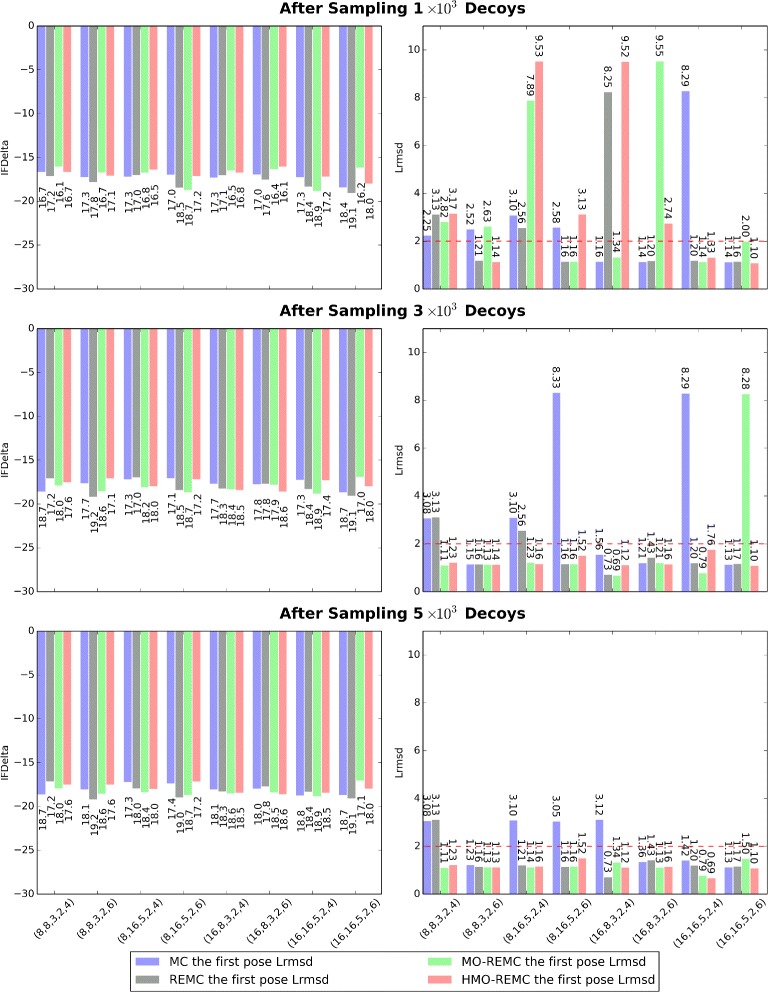
For 2PRG, summaries of the IFDelta and Lrmsd using different initial parameters, sampling methods, and scales

Finally, we present further evidence that the MO-REMC and HMO-REMC sampling methods are effective sampling techniques in temperature space. For 2PRG, we conduct more than one simulation run using different initial parameters((*numR,numC,repackNth,minT,maxT*) : = (8, 8, 3, 2, 4), (8, 8, 3, 2, 6), (8, 16, 5, 2, 4), (8, 16, 5, 2, 6), (16, 8, 3, 2, 4), (16, 8, 3, 2, 6), (16, 16, 5, 2, 4), and (16, 16, 5, 2, 6), respectively.). In this way, the summaries of IFDelta and Lrmsd through creating different trajectories(decoys) of configurations for each initial parameter are compared in Fig. 9. We show that using different initial parameters, the new methods (including MO-REMC and HMO-REMC) proposed in this research can efficiently converge to possible near-native solutions. In addition, we also compare the necessary number of sampling decoys to reach convergence in simulation runs using different initial parameters. The results show that when using different initial parameters, better near-native conformations can be achieved after sampling 5 ×10^3^ decoys(*numR*×*numC*×*N* MC steps, the *N* is the number of decoys). However, for different initial parameters, the necessary number of sampling decoys may be conspicuously different when using different sampling methods.

## Conclusions

In this study, we developed REMC sampling methods based on multi-objective optimization for predicting conformations in protein–small ligand docking with RosettaLigand. We used temperature replica exchange to enhance conformational sampling between Pareto optimal solutions, and the concept of non-dominated solutions was applied to solve the replica selection problem in our REMC enhanced sampling methods. In contrast to most other MC and REMC methods, the MO-REMC method selects non-dominated solutions, which depend on archived solutions measured in terms of the objective MC steps and TScore values, in order to find a set of similar replicas with lower energy conformations but that are also as diverse as possible. The MO-REMC and HMO-REMC methods achieve better integration of the REMC sampling method and critical conformation structures of the current sampling state. Using a benchmark data set of 16 protein-ligand test cases with different chain lengths in terms of amino acids, we assessed various comparison measures, i.e., TScore, IFDelta, and Lrmsd. We also considered the funnel-like character of the energy landscape, the probability of finding each replica at different temperatures, and the rate of convergence in the TScore, IFDelta, and Lrmsd scores.

For the targets tested in our benchmark data set, we found that the ligand pose was correctly positioned within 2Å Lrmsd for 11/16 of these targets using the HMO-REMC sampling method after 1.28 ×10^6^ MC steps. The performance of the proposed MO-REMC sampling method was better than that of the MC and REMC methods in most cases, whereas MC generally performed better but converged slowly. The MO-REMC sampling method achieved significantly faster convergence of the lower energy poses and identified more correct docked complexes with near-native decoys than the MC and REMC sampling methods. Moreover, the results showed that HMO-REMC obtained faster convergence and more distinct solutions than MO-REMC in each run for most of the targets. The MO-REMC and HMO-REMC methods required the same or slightly more time than MC and REMC for the same number of sampling steps. Moreover, for the 1DBJ, 1JD0, 1K3U, 1PQ6, 1Y6B, 2PRG, 4TIM, 6TIM, and 7CPA targets, the performance of HMO-REMC was much better than that of MO-REMC. The HMO-REMC sampling method captured much of the possible variation in the conformations for most of the test cases, and it also sampled lower binding energy conformations within an Lrmsd of 2Å for the conformations of these targets. The HMO-REMC hybridizes two scenario combinations for the Pareto optimal solutions with the ranking-based MO-REMC method and it worked well for many targets. An interesting feature of the MO-REMC method compared with other REMC algorithms is that many non-dominated solutions are chosen as the current replicas for exchange. Thus, sampling at a lower energy is a much more greedy process, which leads to better protein–ligand conformational sampling performance. Clearly, this feature can also be incorporated in addition to the concept of Pareto front solutions in other ensemble-based sampling methods in order to improve their performance. In addition, experimental results showed that faster convergence to the global optimal solution does not necessarily provide an efficient algorithm for enhancing conformational sampling of the phase space. Use of temperature replica exchange to enhance conformational sampling between non-dominated solutions can also provide good convergence of the available conformational space including available near-native structures.

In the future, the proposed MO-REMC method may be extended in several ways. Even though detailed balance is not satisfied in the MO-REMC and HMO-REMC sampling methods, some balance condition may still be efficient if it is proved that it provides a good sampling method. We can still generate an algorithm that may satisfy the balance criteria, for example, instead of selecting the conformation ensemble of Pareto optimal solutions, the configurations to be swapped from the history archives can be randomly selected instead. Moreover, in order to obtain performance improvements, several enhanced sampling techniques, including Hamiltonian replica exchange and well-tempered ensemble approaches, or even a dynamic temperature selection strategy, can be incorporated in the MO-REMC and HMO-REMC methods.

## Abbreviations

HMO-REMC: Hybrid MO-REMC
IFDelta: Binding energy interface delta
Lrmsd: Ligand of RMSD
MC: Monte Carlo
MO-REMC: Multi-objective optimization-REMC
MOP: Multi-objective optimization problem
REMC: Replica exchange Monte Carlo
TScore: The RosettaLigand energy function total score
